# Comparative MicroRNA Expression Profiles of Cynomolgus Monkeys, Rat, and Human Reveal that miR-182 Is Involved in T2D Pathogenic Processes

**DOI:** 10.1155/2014/760397

**Published:** 2014-11-04

**Authors:** Jinghui Zhou, Yuhuan Meng, Shuai Tian, Junhui Chen, Mingyu Liu, Min Zhuo, Yu Zhang, Hongli Du, Xiaoning Wang

**Affiliations:** ^1^School of Bioscience and Bioengineering, Guangdong Provincial Key Laboratory of Fermentation and Enzyme Engineering, South China University of Technology, Guangzhou 510006, China; ^2^Guangdong Key Laboratory of Laboratory Animals, Guangzhou 510663, China; ^3^Chinese PLA General Hospital, Beijing 100853, China

## Abstract

Type 2 diabetes (T2D) is a prevalent disease that happens around the world and usually happens with insulin resistance. MicroRNAs (miRNAs) represented important roles in the suppression of gene expression and were proven to be related to human diseases. In this study, we used cynomolgus monkey fed with normal and high fatty diet (HFD), respectively, to analyze the miRNA expression profile in whole blood by deep sequencing. Finally in total 24 miRNAs with differential expression were filtered. Among them, miR-182 related to the insulin resistance by modulating *FOXO1* and PI3K/AKT cascade and had the greatest copy number in the whole blood. Decrease of miR-182 in T2D cynomolgus individuals is completely consistent with the previous studies in human and rat. Integrating miR-182 tissue expression profile, target genes, and copy number in blood reveals that miR-182 plays a key role in crucial genes modulation, such as *FOXO1* and *BHLHE22*, which leads to potential hyperglycemia and modulates the insulin secretion. In addition, miR-182 might regulate the processes of both cell proliferation and apoptosis that play crucial role in determining the cells' fate. Therefore, miR-182 can be a biomarker in diagnosis of the potential T2D that has benefits for medical purpose.

## 1. Introduction

Type-2 diabetes (T2D), characterized by insulin resistance of target tissues and insufficient insulin secretion from pancreatic beta cells, is a prevalence disease that happened all around the world in high frequency. The etiology of T2D is complex. It cannot be explained by genetics alone, as the environmental factors and the adult-lifestyle behaviors also contribute significantly [1].

In the 1993, since Lee and coworkers discovered the small RNA so-called microRNA (miRNA) which can regulate the expression level [2], researchers worldwide began to focus on it and relevant studies continuously happened. miRNA is a kind of noncoding RNA in the species with the endogenous length of 20–23 nucleotides. It induces degradation or represses translation of the mRNA by complementary pairing to the target mRNA and thereby makes negative regulation to the gene expression at the transcript level [3]. In these years, miRNA was proved to have possibility in regulation of the physiological and pathological processes [4].

Recent studies demonstrated that miRNAs play significant roles in T2D. For the in-depth study of the mechanism about how miRNAs regulate the T2D relevant tissues and about the relationship between miRNA and T2D, numerous studies were done on the platform of T2D patients and animal models. Moreover, the technologies in the miRNA analysis usually focus on the microarray which may lose the important information or induce false positive frequently. The animal models used in the recent studies mainly focused on rats or mice and found that the miRNAs can be related to T2D, obesity, and inflammation in the tissues and organs including pancreas islet, skeletal muscle, adipose tissue, blood, *β*-cell, and liver [5–10].

The miRNAs in the circulating blood can be used for diagnosis of diseases, because they can reflect the injury which happened in the tissues or organs to some extent. The miRNAs can be preferentially produced in some tissues and usually at low concentration in the plasma. During the disease, for the tissue injury, some differential expressed miRNAs can be released into the circulating blood fluid and can be detected by the blood-based assay. Therefore, depending on this principle, it becomes possible to diagnose the diseases according to the circulating miRNAs [11]. In this study, we used a T2D animal model of cynomolgus monkey (*Macaca fascicularis*) for the miRNA profile analysis for the first time. Moreover, the animals were divided into intact T2D monkey and high fat diet (HFD) T2D monkey to evaluate miRNA varieties in circulating blood tissue by the deep-sequencing analysis which can provide defined copy number. Compared with the previous method such as microarray, the deep-sequencing can provide the exact copy number of all the miRNAs in one experiment. Thus, it provides possibility to represent the comprehensive regulation mechanism in the specific tissues [12–14]. By analyzing the results and comparing them with the previous studies, we attempt to explain the potential miRNA regulation network during T2D.

## 2. Materials and Methods

### 2.1. Materials Preparation and Deep Sequencing

The animals were handled according to the NHP (nonhuman primates) policy. All macaque experiments were subjected to approval and surveillance by the Institutional Animal Care and Use Committee of Guangdong Landau Biotechnology Co., Ltd. In total, 150 cynomolgus monkeys of more than 8 years old were prepared and fed differently: 100 animals were fed with regular monkey chow and 50 were fed with monkey Chow of high fat (contributed 40% energy to the Chow). The animals were fed in groups in the place with environmental enrichments such as toys and plants. Only when we fed the animals or extracted the blood, the animals would be moved to the cages for short time. Animals were fed with apples (100 g/day each) and monkey chow (Feed Research Institute, Guangzhou, Guangdong) twice daily and allowed free access to water. Cynomolgus fed with regular monkey chow were considered as intact individuals, and those fed with monkey chow of high fat were considered as HFD individuals. Fasting glucose, glycated hemoglobin, fasting insulin, HDL-ch, TG, LDL-CH, and CHOL of all animals were monitored more than 5 times and each time per 30 days by the OneTouch UltraVue (Johnson Company, USA), Adamstma1c-8160 (Arkray Company, Japan), and CardioChek lipid instrument (Polymer Technology Systems, Inc., America), respectively. The case with T2DM was defined as of fasting blood glucose of over 108 mg/dL and glycated haemoglobin of more than 6% according to the previous studies [15, 16]. The T2D individuals were determined based on the physical indexes of the last consecutive 5 times. As soon as the monkeys reached the standard of T2D, the blood samples were extracted. Blood samples of 12 cynomolgus monkeys (including 6 Intact and 6 HFD individuals, resp.) were collected from Landao Biotechnology Co., Ltd., (Guangdong, China) and stored at −80°C with the TRIzol reagent (Invitrogen, USA). During the period of blood extraction, no anesthetic was used.

The small RNA libraries were constructed following the manufacturer's instructions for the Small RNA Sample Prep Kit (Illumina). Briefly, total RNA was isolated with TRIzol reagent from blood samples, and the small RNAs were ligated with adapters followed by reverse transcribe and amplification. The PCR products derived from the 22 nt and 30 nt small RNA fragments were purified from 6% Novex TBE PAGE Gel. Purified microRNAs were sequenced on the Illumina Genome Analyzer IIx for 36 cycles, and sequencing was performed at Shanghai Biotechnology Corporation.

### 2.2. miRNA Expression Profile

The adaptor sequences were trimmed from the raw reads after deep sequencing. The reads were extracted if they reached the criteria of being without unidentified base, having base quality greater than 10, counts of the unique reads over 10, and length between 18 nt and 35 nt. The output reads were matched to the mature miRNA of the miRbase (release 20) by the software Bowtie (version 0.12.9) [17]. The reads which could match the mature miRNA region ±4 nt at the 5′-end and 3′-end were therefore accepted (allowing one base mismatch) [12]. The numbers of the reads categorized in the same miRNA were summed up, and then the copy number of each miRNA in 12 samples was therefore obtained.

The copy number of each miRNA could be used to estimate expression level of that miRNA. Before the assay of detecting the significant expression differences, the process of normalization is important for eliminating the bias between different samples. For doing the normalization, the sample of the individual with the greatest total number of valid reads was used as the reference. Then in other samples, the copy numbers of each miRNA were divided by the scaling factor ((total number of valid reads, given)/(total number of valid reads, reference)) [12]. Therefore, the normalized copy numbers of each miRNA in 12 samples were obtained.

In this study, the monkeys fed with normal diet were called intact monkeys, while those fed with HFD were called HFD monkeys. We built up three measurements for comparing: (1) All comparison: T2D (including intact-T2D and HFD-T2D individuals) compared to no-T2D (including intact-normal and HFD-normal individuals); (2) intact comparison: intact-T2D individuals compared to intact-normal individuals; (3) HFD comparison: HFD-T2D individuals compared to HFD-normal individuals. To illustrate the influence of HFD separately, the extra compare group (no-T2D compare) was set up including the no-T2D individuals in normal diet and HFD. In each comparison, the copy numbers of the samples were used to estimate the significant difference of the miRNA expression.

The significant differences of the miRNA expression were determined by the fold change and *P* value statistic. In the former one, considering some miRNAs expressed with very low copy number, we added a given low number of 40 units to each miRNA (e.g., (2000 + 40)/(1000 + 40) = 1.96~2) [12]. The miRNAs with fold change value greater than 2 were filtered. In *P* value statistic, we did the two-sided Student's *t*-test and accepted those miRNAs with the *P* value lower than 0.05. Those miRNA that reached each of the criteria above would be regarded as significant differences of expression.

Because the type I error might happen due to the multiple groups of data, the ANOVA test has been performed for the further verification of the filtered miRNAs. All the processes of ANOVA were performed on the software R. In the All comparison, the samples would be separated into two levels for the two-way ANOVA analysis, depending on diet conditions and whether they suffered from T2D. The variance of each miRNA would be checked for homogeneity of variance by Bartlett's test [18]. If the variance was not in homogeneity, the copy number of the miRNA would be transformed into log⁡⁡2 value for modification. The results of the ANOVA test would be convincing only when the variance was in homogeneity (*P* value > 0.05). In the two-way ANOVA test, the copy numbers of each miRNA from 12 samples were analyzed. In the rest of the three comparisons, the one-way ANOVA was performed. The corresponding results to the four comparisons (All comparison, intact comparison, HFD comparison, and no-T2D comparison) were extracted. The acceptance criteria would be *P* value lower than 0.05.

## 3. Results

### 3.1. Cynomolgus Monkeys Used as Animal Model in T2D miRNA Analysis

For the miRNA study, cynomolgus monkeys were prepared and fed differently. We divided the monkeys into four groups depending on the diet conditions and whether they suffered from T2D. The monkeys fed with normal diet and kept healthy were regarded as intact-normal monkeys, whilst those with normal diet but spontaneously got sick in T2D were regarded as intact-T2D monkeys. Similarly, the monkeys fed with high fatty diet can be divided into HFD-normal and HFD-T2D monkeys. Finally 12 monkeys in accordance with the physiological indexes (Table 1), 3 monkeys in each group, were chosen for the miRNA expression profile analysis by deep sequencing.

### 3.2. miRNA Profile by Deep Sequencing Revealed High Credibility after Genome Mapping

Following the processes of deep sequencing, there were in total 147,251,331 raw reads outputted by the Illumina Genome Analyzer IIx. After trimming the adaptor sequences, the raw sequence reads were firstly filtered based on base quality (*Q* ≥ 10), which we called the clean reads (~99%). The second filter was done depending on the length of the reads between 18 and 35 nt, and the counts of each reads are not fewer than 10. Moreover, all the reads which contained unidentified base (N) were deleted. Finally, 93.78% of the raw reads remained and defined as valid reads. The details were listed in Table S1 (see Supplementary Material available online at http://dx.doi.org/10.1155/2014/760397).

All the valid reads were mapped to the human and cynomolgus monkey genomes [19, 20]. The similarity mapping ratios (~93.6%) indicated that the valid reads are convincible and appropriate for study (Table S1). Most of the valid reads (~93%) could be mapped to the mature miRNA sequences in miRBase v20 [21], and minority valid reads (~2%) could be mapped to the ncRNA database except microRNA in Ensembl v73 [22]. The details were also listed in Table S1. The results also obviously suggested that the majority of the reads of cynomolgus monkeys showed high similarity to the human genome and also could be found in the current miRNA databases.

### 3.3. Differential Expression miRNAs under Different Diet Conditions

After matching the human mature miRNA database and summing up the copy numbers of each kind of the miRNAs, there were 360 unique miRNAs identified in 12 samples. The detailed expression profile of each sample after normalization was shown in Table S2.

The differentially expressed miRNAs between control and T2D groups were compared by three measurements: All comparison, intact comparison, and HFD comparison (see Section 2). The criteria used for filtering the differential expressed miRNAs was *P* value lower than 0.05 (by two-sided Student's *t*-test) or the modified fold change value greater than 2. Finally, 24 differential expressed miRNAs were filtered (Table S3).

Among the 24 specific miRNAs, 13 of them, such as miR-182/196a/381/499a/99a [6], miR-183 [6, 23], miR-409 [23], miR-146b [6, 24], miR-143 [6, 24], miR-148a [24, 25], miR-204 [5], and miR-9 [6], have been reported to involve in T2D process in mouse or rat. The other miRNAs which have not been reported in mouse or rat model might be due to the species or tissue specificity. Thus, cynomolgus macaque monkey, which is similar to mouse and rat, could be an available animal model in the T2D research.

**Figure 1:**
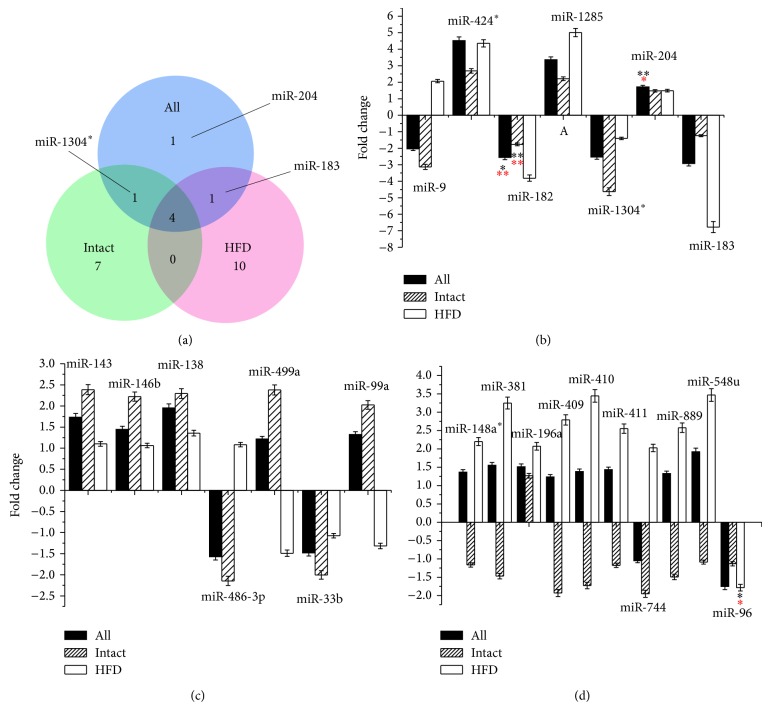
The general expression tendencies of the 24 specific miRNAs in All comparison, intact comparison, and HFD comparisongroups. The comparisons were carried out following the criteria where T2D individuals were compared with no-T2D individuals. All comparisons were carried out in all the monkeys without considering the diet conditions, while intact comparison was only in intact monkeys and HFD comparison was in HFD monkeys. (a) The Venn diagram represents the numbers of differential expressed miRNAs in All comparison (blue), intact comparison (green), and HFD comparison (red) groups. (b) The 7 specific miRNAs in the All comparison groups. All the miRNAs are in the same expression tendencies except miR-9. The positive values of the fold change mean that the miRNAs are upregulated in the T2D samples, whereas the negative values mean the downregulated expression. (c) The specific 7 miRNAs uniquely existed in the intact comparison group. Without miR-486-3p, miR-499a, and miR-99a in inconsistent expression between intact and HFD groups, others are in the same expression tendencies. (d) The 10 specific miRNAs uniquely existed in the HFD group. All of the miRNAs, except miR-196a and miR-96, are in inconsistent expression tendencies between the intact and HFD induced groups. Statistically significant differences are tested at *P* < 0.05 significance. The significance in *t*-test was labeled in black color, whilst that in ANOVA test was in red color (*P* ^*^ < 0.05, *P* ^**^ < 0.01).

The relationships of the miRNAs between three comparisons were shown in Figure 1(a). Four microRNAs, including miR-9, miR-1285-3p, miR-424-3p, and miR-182-5p, were filtered in all three comparisons. They might involve in both T2D with normal diets and HFD. The expression inclinations with fold change values of each of the miRNAs were shown in the Figures 1(b), 1(c) and 1(d). Among them, in the HFD comparison, we noticed that there were 8 miRNAs with the inconsistent expression tendencies (Figure 1(d)). It suggested that the expression of most miRNAs filtered in the All and intact comparisons was less affected by the diet condition, whereas the expression of the other miRNAs with the inconsistent expression tendencies was affected by HFD significantly. Based on the physical indexes, it is in the most possibility that, while the internal cholesterol level comes to be extremely high, the miRNAs would more likely come to have opposite expression tendencies which could be inconsistent with those in All and intact groups. Therefore, it contributes to the differences of molecular mechanism between the normal diet and HFD individuals in T2D.

**Figure 2:**
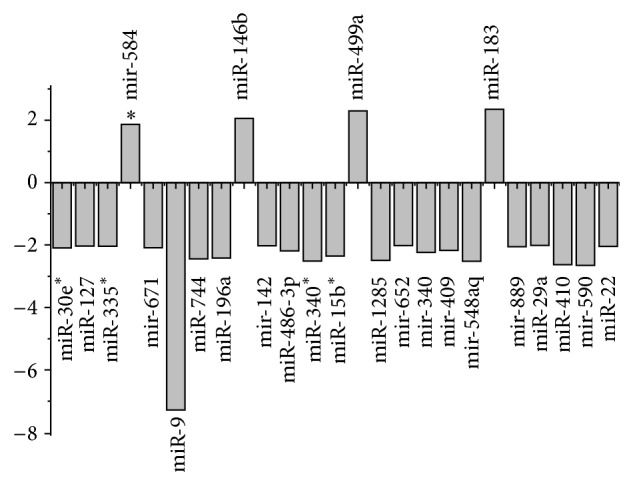
The comparison between the no-T2D individuals in normal diet and HFD. The fold-change of each miRNA in no-T2D comparison was represented.

To illustrate the influence of the HFD and the relationship between HFD and T2D, we compared the microRNA expression level of intact normal monkeys to those of HFD-normal monkeys (no-T2D comparison). The results were summarized in Figure 2. Integrating the results of Figure 1, the miR-146b, miR-486-3p, and miR-499a, which were detected both in the intact comparison and no-T2D comparison but not in HFD comparison, might involve both the progresses of T2D fed with normal diets and fat metabolism fed with HFD. The microRNAs filtered in intact comparison but not filtered in HFD comparison and no-T2D comparison, including miR-143-3p, miR-99a-5p, miR-138-5p, miR-1304-3p, and miR-33b-5p, might involve the progress of T2D fed with normal diets only. The miR-381-3p, miR-548u, miR-411-5p, miR-148a-5p, and miR-96-5p which were filtered in HFD comparison but not in intact comparison and no-T2D comparison might involve the progress of T2D fed with HFD only. The rest of the miRNAs were filtered in no-T2D comparison but not in the other three comparisons which might be related to fat metabolism fed with HFD only.

### 3.4. miR-182 as a Crucial miRNA in the T2D Individuals

It can obviously be observed that the miR-182 was in the significant differential expression in the All, intact, and HFD groups, especially in the All compare that reached the entire criteria (*P* < 0.05 in *t*-test and ANOVA test, fold change >2). Moreover, the downregulated expression of miR-182 is consistent with the previous study in the blood plasma samples of human and rat [6] and the microarray experiment in peripheral blood with lean and obese human (Accession number: GSE27645; significance was calculated by *t*-test). In addition, as the miRNA in the circulating blood usually come from the organs and the impairment of the tissues, the blood miRNA can be reflective to those tissues to some extent [11, 26]. Therefore, the tissues and organs which detected the differentially expressed miR-182 in the previous studies and the valid target genes of miR-182 were summarized and shown in Table 2. The references which provided the valid target genes of miR-182 were listed in Table S4. By using the functions of Ensembl, NCBI, and TiGER databases [27], the tissue specificity of the target genes was identified. Most of the target genes are commonly expressed in the organs and tissues listed in Table 2. The details of the tissue specificity of the target genes were summarized in Table S4. According to Table 2, the differentially expressed miR-182 detected in the circulating blood can also be detected in the peripheral organs such as adipose, liver, muscle, and pancreas. Moreover, the downregulated expression of miR-182 was represented consistently in these tissues as well. Therefore, it can be obviously noticed that miR-182 is closely related to T2D and might influence or lead to some of the syndromes during T2D.

## 4. Discussion

In this study, the cynomolgus monkey models in normal diet and HFD have given evidence of some of the miRNAs that can influence the process of T2D. For instance, the up-expressed miR-143 and miR-138 can, respectively, target the genes,* HK2* and* HK1*, which are the crucial enzymes in glycolysis and so that lead to potential glycemia [28, 29]; miR-9 and miR-204 were reported that they can regulate the insulin secretion by targeting the gene,* SIRT1* [30–32], while miR-96 can decrease the expression of* NOC2* which is involved in the insulin secretion [33]. Those reported differential expressed miRNAs proved that the results in our study can be convincing and the models are also available.

**Figure 3:**
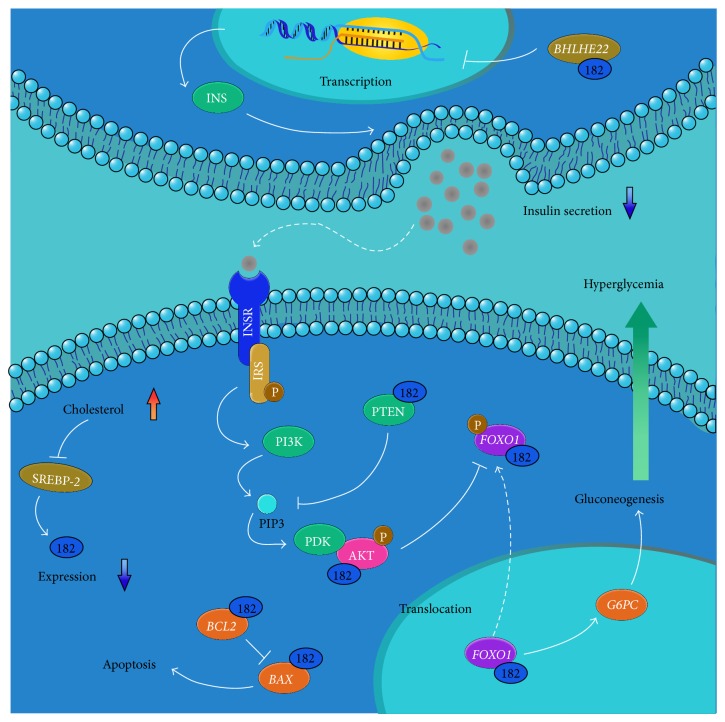
Putative regulation network of miR-182 related to the syndrome in T2D. The miR-182 can be linked to the syndrome of T2D according to the valid target genes analysis. The crucial genes and pathways that related to insulin resistance, insufficient insulin secretion, and the impairment of the tissues were represented in the figure.

In our results, miR-182 is in conserved downregulated expression across the species such as human, rat, and cynomolgus macaque. Therefore, it might have close relationship with T2D. However, currently few reports were focus on the relationship between miR-182 and T2D. By analyzing the target genes of miR-182, the underlying mechanism can be deduced which give us hints to understand about miR-182's functions and relationship to T2D. The general putative regulation network is summarized in Figure 3.

### 4.1. The miR-182 Can Be a Reliable Biomarker for Diagnosis of T2D

In our study, miR-182 was in the highest copy number among the whole differentially expressed miRNAs. In the intact and HFD comparison groups, it is noteworthy that miR-182 represented down-expression consistently (both were almost in 2-fold change). Moreover, in the T2D individuals, the miR-182 in the HFD group was expressed in much lower level than that in the intact group (Table S3).

Such phenomenon may be caused by the high cholesterol level in the organisms. From the previous study, the miR-96/182/183 cluster is regulated by SREBP-2 which is a function as it activates the expression of many key genes for cholesterol level and uptake. While the cholesterol gets higher, the miR-182 and SREBP-2 expression level will tend to be lower. From Table 1, as the cholesterol level was even higher in T2D than healthy individuals, the miR-96/182/183 would accordingly come to be downexpressed [34]. Such case was also proved by the expression level of miR-33a which is usually expressed followed with SREBP-2 [35–39], as miR-33a was in low copy number (<20, data not show in the paper). As described at Table 1, comparing T2D to healthy individuals, in the intact group, the mean cholesterol content was increased about 0.16 mmol/L which is less than those in the HFD group 1.00 mmol/L. Thus, such case may contribute to the greater fold change of miR-182 in HFD group than intact group (Figure 1). Interestingly, by comparing the intact-normal and HFD-normal individuals, we found that miR-182 was not in significant expression (Figure 2). In another word, the expression of miR-182 was generally stable in both groups. The ANOVA test also proved that, in the expression of miR-182, the interaction between HFD and T2D was not significant (*P* value = 0.24). Therefore, it cannot simply be said that the level of miR-182 is only related to the cholesterol level. In the healthy individuals, there might be some unknown mechanisms to modulate the miRNAs level. Thus, some important miRNAs including miR-182 can be kept stable so that they cannot get into the endocrinal dysfunction and therefore protect the internal environmental balance. While the individual gets sick in T2D, the balance of the internal environment can get into dysfunction and the miR-182 therefore becomes downexpressed which influences the process of T2D. Therefore, because of the characteristics of miR-182 that keeps stable in the healthy individuals but downexpressed in the T2D period no matter in the lean or obese individuals, it comes to be a reliable biomarker for diagnosis of T2D.

### 4.2. The Syndrome Which Occurs during the T2D Can Be Potentially Linked to the miR-182

The relationship of the miR-182 and the mechanism linked to T2D was summarized in Figure 3. From the previous studies, we supposed that the miR-182 can influence the process of T2D to modulate the insulin secretion, attenuate the effect of insulin signaling pathway, and do the possible regulation of the cell's fate. These hypotheses were further elaborated in the following.

Liver and skeletal muscle tissues have an important function in glucose metabolism. During the period of T2D, the glucose level in the peripheral blood can become even higher than the healthy individuals. From the previous studies, it is known that the expression of the gene* Foxo1* is under the modulation of the miR-182 [40, 41]. In this case, the serious downexpression of miR-182 (~2-fold in intact group and ~4-fold in HFD group) might finally result in increasing ofmRNA level of* FOXO1*. Moreover, it has been reported that the mRNA level of* FOXO1* is increased during T2D which proves this hypothesis [42]. FOXO1 can increase the transcription of the gene downstream such as* G6PC* that takes part in the process of gluconeogenesis. Therefore, it is in high possibility that serious downexpression of miR-182 increases expression of* FOXO1* which contributes to hyperglycemia in diabetes [43, 44].

Insulin resistance is a common symbol of the T2D. Generally, the PI3K-AKT cascade performs important function to react to insulin signal. The PI3K-AKT-FOXO1 signaling usually functions to the phosphorylation of FOXO1. The activated AKT can phosphorylate FOXO1 and thereby promotes FOXO1's nuclear to cytoplasmic translocation and degradation [45–47]. By observing the target genes of miR-182, the gene* PTEN* is under its suppression [48, 49]. The PTEN can inhibit the function of PIP3. Thus, the PI3K-AKT cascade can be disturbed in order to attenuate the insulin signal. In this case, the phosphorylation of the AKT can be attenuated so that the inactivated AKT cannot perform the function to FOXO1 and lead to hyperglycemia. As the miR-182 has downregulated expression that the PTEN can express higher level than usual, the upgoing expressed PTEN will attenuate the signal of insulin that come to the potential insulin resistance. Therefore, as in the HFD group, in miR-182 expressed lower than that in intact group, the individuals may be in more serious insulin resistance. In this case, the insulin secretion would be stronger for compensating the need of insulin (Table 1).

In addition, the insulin secretion happens in the pancreas *β* cell. Currently, report has shown that miR-182 is in the positive regulation toward insulin secretion by inhibiting the expression of* BHLHE22* which is a molecule that can downregulate insulin mRNA transcription in islet [50]. From Table 1, it can be obviously observed that comparing HFD group with intact group, the insulin content came to be higher as more glucose was absorbed from the high fatty and fructose diet. However, while comparing the insulin content of T2D and healthy individuals, no matter in HFD group or intact group, only just few differences were represented. Such phenomenon may be explained as the miR-182 was downregulated during T2D and hence causing the insulin mRNA transcripts to decrease. Therefore, the insufficient insulin secretion becomes hard to stabilize the glucose level during T2D.

Finally, during the T2D, the impairment of the pancreas commonly occurred in the T2D individuals [51]. It may be linked to the differential expression of miR-182 which enforces the process of apoptosis, because it can be noticed that the miR-182 may be linked to the two opposite processes of cell proliferation and apoptosis. Two genes,* BCL2* and* BAX*, that led to the processes of antiapoptosis and apoptosis are under suppression of miR-182, respectively [52, 53]. In addition, some of the genes such as* MYC* which is important in the cell cycle regulation [48, 49] and* RELA* which is in the functions of inflammatory reaction and antiapoptosis are also regulated by miR-182 [54]. Thus, the fate of the cell can be determined by the miR-182 but the underlying mechanism about how to regulate which functions it would perform is still unknown.

In the general view, miR-182 represents a crucial component in the modulation of the molecules during T2D. It takes part in the process of insulin signaling and insulin secretion. Thus, the importance of this miRNA is expected to be a biomarker in diagnosis of T2D. Moreover, as the miR-182 showed close relationship to the T2D syndrome, regulating the expression level of miR-182 might be a possible strategy of treating T2D. Therefore, further studies to clarify the functions of the miR-182 might be needed for the medical purpose in diagnosis of T2D and therapy development toward T2D.

## 5. Conclusion

In this study, we used the cynomolgus macaque as T2D animal model for doing the miRNA profile analysis for the first time and finally got the same result of differential expressed miR-182 toward the previous experiments done by human and rat. Moreover, the symbol of downregulated expression of miR-182 is consistent whether in the blood sample or the peripheral organ samples. Thus, miR-182 might be in close relationship to T2D. However, in the recent years, little attention was paid to the relationship between miR-182 and T2D. It might be due to the limitation of the techniques such as microarray which usually accompanied with high false positive rate that may lose important information. Therefore, we used the deep sequencing which can provide the defined copy number of miRNA so that the results can be more convincing. In addition, we divided the animal samples into the normal diet and high diet group which can provide general view of the idea that miR-182 is conserved, downregulated, and expressed in any diet conditions. By the further valid target genes analysis, it is represented that the miR-182 has potentially an important role in the pathogenic process of T2D including the insulin resistance, insulin secretion, and the cell apoptosis which potentially cause tissue impairment. In this case, the downregulated miR-182 is may possibly become a biomarker in detecting the potential T2D which makes benefits to the medical development.

## Supplementary Material

Table S1. The detailed information of the reads filtering and mapping.
Table S2. The miRNA expression profile.
Table S3. The fold change value and *P*-value of the filtered miRNAs in the four comparisons.
Table S4. The references and specific expressed tissues of the valid target genes of miR-182.

## Figures and Tables

**Table 1:** The physical indexes of the cynomolgus macaques.

No.	Sex	Age (year)	Weight (kg)	GLU^a^ (mmol/L)	HBA1C (%)	Fasting insulin (uU/mL)	HDL-CH (mmol/L)	TG (mmol/L)	LDL-CH (mmol/L)	CHOL (mmol/L)	Comment
47	M	19	8.2	22.6 ± 2.9	10.0 ± 0.6	26.0 ± 3.0	1.1 ± 0.1	1.2 ± 0.1	1.7 ± 0.1	2.8 ± 0.2	Intact T2D
64	M	18	6.9	17.0 ± 1.4	12.4 ± 1.5	7.7 ± 1.9	0.9 ± 0.1	2.1 ± 0.2	1.4 ± 0.7	2.9 ± 0.7	Intact T2D
8	F	22	5.3	7.9 ± 1.8	6.1 ± 0.2	44.1 ± 11.0	1.3 ± 0.1	1.1 ± 0.0	1.1 ± 0.1	2.3 ± 0.4	Intact T2D
52	M	18	9.2	2.8 ± 0.6	4.0 ± 0.0	26.9 ± 2.1	1.6 ± 0.0	0.3 ± 0.0	1.0 ± 0.1	2.5 ± 0.0	Intact
61	M	17	7.7	2.8 ± 0.6	4.1 ± 0.0	15.9 ± 12.7	1.4 ± 0.1	0.4 ± 0.3	1.1 ± 0.0	2.4 ± 0.1	Intact
1	F	18	6.1	3.5 ± 0.31	4.6 ± 0.2	30.7 ± 11.2	1.4 ± 0.0	1.3 ± 0.2	1.0 ± 0.3	2.5 ± 0.2	Intact
11	M	12	11.1	8.6 ± 4.3	8.5 ± 0.1	356.1 ± 62.9	0.7 ± 0.2	4.4 ± 0.3	1.6 ± 0. 3	4.0 ± 0.5	HFD T2D
25	M	9	11	12.7 ± 0.8	10.8 ± 1.6	229.2 ± 73.6	1.7 ± 0.1	1.1 ± 0.3	3.4 ± 1.8	5.0 ± 1.8	HFD T2D
9	M	18	11.8	7.6 ± 1.5	6.7 ± 0.7	125.2 ± 78.4	1.8 ± 0.3	2.3 ± 0.2	2.6 ± 0.2	4.3 ± 0.6	HFD T2D
31	M	12	11.5	3.2 ± 0.0	4.65 ± 0.2	361.9 ± 153.4	1.6 ± 0.1	2.1 ± 0.2	1.5 ± 0.2	3.6 ± 0.3	HFD
34	M	13	9	3.1 ± 0.2	3.9 ± 0.1	107.8 ± 8.1	1.9 ± 0.0	0.4 ± 0.1	1.3 ± 0.0	3.0 ± 0.0	HFD
6	F	21	6.5	4.6 ± 0.4	4.6 ± 0.3	115.6 ± 56.4	1.8 ± 0.1	0.7 ± 0.0	1.8 ± 0.2	3.7 ± 0.5	HFD

^a^GLU: glucose; HBA1C: glycosylated hemoglobin A1c; HDL-CH: high density lipoprotein cholesterol; TG: triglyceride; LDL-CH: low density lipoprotein cholesterin; CHOL: cholesterol.

**Table 2:** The details of the differential expressed miR-182 in the T2D individuals.

Species and tissue	Fold change (T2D/normal)	*P* value	Reference or accession ID
Human blood (lean)	−1.3956	0.2651	GSE27645
Human blood (obese)	−1.1258	0.7116	GSE27645
Human blood	−2.5800	0.0400	[6]
Rat blood	−1.8560	0.0050	[6]
Rat adipose	−3.5220	0.0010	[6]
Rat pancreas	−1.2590	0.0020	[6]
Rat skeletal muscle	−4.2260	0.0230	[6]
Rat liver	−2.6130	0.0110	[6]
Cynomolgus blood (All comparison)	−2.5629	0.0224	—
Cynomolgus blood (intact comparison)	−1.7684	0.0047	—
Cynomolgus blood (HFD comparison)	−3.8130	0.1645	—

**Valid target genes of miR-182**	*ADCY6, AKT1, ARRDC3, ATOH1, BAX, BCL2, BCL2L11, BDNF, BHLHB5, BRCA1, CASP2, CCL27, CCND1, CCND2, CD38, CDC42, CDKN1A, CLOCK, COX8A, CREB1, CTTN, CYLD, DICER1, DNTT, DOK4, E2F3, EGFR, EGR1, EIF2C1, EIF2C2, EIF2C3, ENPP3, EP300, ERBB2, EVI1, FBXW7, FOXF2, FOXO1, FOXO3, FRAP1, HBEGF, HMGA2, IL17A, IL17F, IL2, IRAK2, JAK2, KIT, MITF, MOCOS, MOS, MTSS1, MYB, MYC, MYCN, MITF, NAMPT, NEUROD4, NFIB, NRIP1, PAK1, PAK3, PCNA, PDCD4, PFN1, PNLIP, PRB1, PRMT5, PTEN, RAC1, RARG, RDX, RECK, RELA, RGS17, RNASEN, ROS1, SAG, SIRT1, SLC12A2, SLC1A1, SLC7A5, SMAD4, SNAI2, SOX6, SSSCA1, TNF, TP53, TSC22D3, TBX1, ZEB1, ZEB2, ZNF828 *		
