# Supplementary material for: Comparative MicroRNA Expression Profiles of Cynomolgus Monkeys, Rat, and Human Reveal that miR-182 Is Involved in T2D Pathogenic Processes
Source: J Diabetes Res. 2014 Nov 4;2014:760397. doi: 10.1155/2014/760397 (PMC4235598; doi:10.1155/2014/760397)
Supplement: Supplementary file 1 — Table S1. The detailed information of the reads filtering and mapping. Table S2. The miRNA expression profile. Table S3. The fold change value and P-value of the filtered miRNAs in the four comparisons. Table S4. The references and specific expressed tissues of the valid target genes of miR-182. [file 760397.f1.zip › Table S4. The summarization of the valid target genes of miR-182.doc]

The valid target genes of the miR-182 were listed below and the corresponding references were summarized and provided. The Tissue-specificity will focus on the crucial tissues that related to T2D, including adipose, liver, muscle and pancreas. It represented that whether the target genes can be expressed in those tissues. The data of the Tissue-specificity was come from TiGER database (http://bioinfo.wilmer.jhu.edu/tiger/), the function of gene expression in Ensembl (http://www.ensembl.org/), and the description in NCBI (http://www.ncbi.nlm.nih.gov/).

\## “No data” means not enough data in the website can explain the tissue-specificity; “N/A” means the gene was not expressed in the crucial tissues.

| **Target Gene** | **Tissue-specificity** | **Ref** | **Target Gene** | **Tissue-specificity** | **Ref** |
| --- | --- | --- | --- | --- | --- |
| *ADCY6* | Adipose, Liver, Muscle, Pancreas |  | *KIT* | Adipose, Muscle, Pancreas |  |
| *AKT1* | Adipose, Liver, Muscle, Pancreas |  | *MITF* | Adipose, Liver, Muscle, Pancreas |  |
| *ARRDC3* | Adipose, Liver, Muscle, Pancreas |  | *MOCOS* | N/A |  |
| *ATOH1* | No data |  | *MOS* | No data |  |
| *BAX* | Adipose, Liver, Pancreas |  | *MTSS1* | Adipose, Liver, Muscle, Pancreas |  |
| *BCL2* | Adipose, Muscle, Pancreas |  | *MYB* | Adipose, Liver |  |
| *BCL2L11* | Adipose, Liver, Muscle, Pancreas |  | *MYC* | Adipose, Liver, Muscle, Pancreas |  |
| *BDNF* | Liver |  | *MYCN* | Adipose, Liver, Muscle |  |
| *BHLHB5* | N/A |  | *MITF* | Adipose, Liver, Muscle, Pancreas |  |
| *BRCA1* | Liver, Muscle, Pancreas |  | *NAMPT* | Adipose, Liver, Muscle, Pancreas |  |
| *CASP2* | Liver, Muscle, Pancreas |  | *NEUROD4* | N/A |  |
| *CCL27* | Pancreas |  | *NFIB* | Adipose, Liver, Muscle, Pancreas |  |
| *CCND1* | Adipose, Liver, Muscle, Pancreas |  | *NRIP1* | Adipose, Liver, Muscle, Pancreas |  |
| *CCND2* | Adipose, Liver, Muscle, Pancreas |  | *PAK1* | Adipose, Liver, Muscle, Pancreas |  |
| *CD38* | Adipose, Liver, Muscle, Pancreas |  | *PAK3* | Liver, Muscle, Pancreas |  |
| *CDC42* | Adipose, Liver, Muscle, Pancreas |  | *PCNA* | Adipose, Liver, Muscle, Pancreas |  |
| *CDKN1A* | Adipose, Liver, Muscle, Pancreas |  | *PDCD4* | Adipose, Liver, Muscle, Pancreas |  |
| *CLOCK* | Adipose, Liver, Muscle, Pancreas |  | *PFN1* | Adipose, Liver, Muscle, Pancreas |  |
| *COX8A* | Adipose, Liver, Muscle, Pancreas |  | *PNLIP* | Liver, Pancreas |  |
| *CREB1* | Adipose, Liver, Muscle, Pancreas |  | *PRB1* | Liver, Muscle, Pancreas |  |
| *CTTN* | Adipose, Liver, Muscle, Pancreas |  | *PRMT5* | Adipose, Liver, Muscle, Pancreas |  |
| *CYLD* | Liver, Muscle, Pancreas |  | *PTEN* | Adipose, Liver, Muscle, Pancreas |  |
| *DICER1* | Liver, Muscle, Pancreas |  | *RAC1* | Adipose, Liver, Muscle, Pancreas |  |
| *DNTT* | No data |  | *RARG* | Liver, Muscle, Pancreas |  |
| *DOK4* | Adipose, Liver, Muscle, Pancreas |  | *RDX* | Adipose, Liver, Muscle, Pancreas |  |
| *E2F3* | Liver, Muscle, Pancreas |  | *RECK* | Adipose, Liver, Muscle, Pancreas |  |
| *EGFR* | Adipose, Liver, Muscle, Pancreas |  | *RELA* | Liver, Muscle, Pancreas |  |
| *EGR1* | Adipose, Liver, Muscle, Pancreas |  | *RGS17* | Liver, Muscle, Pancreas |  |
| *EIF2C1* | Adipose, Liver, Muscle, Pancreas |  | *RNASEN* | Adipose, Liver, Muscle, Pancreas |  |
| *EIF2C2* | Adipose, Liver, Muscle, Pancreas |  | *ROS1* | Adipose, Liver, Muscle, Pancreas |  |
| *EIF2C3* | Adipose, Liver, Muscle, Pancreas |  | *SAG* | Muscle |  |
| *ENPP3* | Liver, Muscle, Pancreas |  | *SIRT1* | Liver, Muscle, Pancreas |  |
| *EP300* | Adipose, Liver, Pancreas |  | *SLC12A2* | Liver, Muscle, Pancreas |  |
| *ERBB2* | Adipose, Liver, Muscle, Pancreas |  | *SLC1A1* | Liver, Muscle, Pancreas |  |
| *EVI1* | Adipose, Liver, Muscle, Pancreas |  | *SLC7A5* | Adipose, Liver, Muscle, Pancreas |  |
| *FBXW7* | Liver, Muscle, Pancreas |  | *SMAD4* | Adipose, Liver, Muscle, Pancreas |  |
| *FOXF2* | Liver, Pancreas |  | *SNAI2* | Liver, Muscle, Pancreas |  |
| *FOXO1* | Adipose, Liver, Muscle, Pancreas |  | *SOX6* | Adipose, Liver, Muscle, Pancreas |  |
| *FOXO3* | Adipose, Liver, Muscle, Pancreas |  | *SSSCA1* | Liver, Pancreas |  |
| *FRAP1* | Adipose, Liver, Muscle, Pancreas |  | *TNF* | N/A |  |
| *HBEGF* | Adipose, Liver, Muscle, Pancreas |  | *TP53* | Adipose, Liver, Muscle, Pancreas |  |
| *HMGA2* | Adipose, Liver, Muscle, Pancreas |  | *TSC22D3* | Adipose, Liver, Muscle, Pancreas |  |
| *IL17A* | No data |  | *Tbx1* | Muscle |  |
| *IL17F* | No data |  | *ZEB1* | Adipose, Liver, Muscle, Pancreas |  |
| *IL2* | No data |  | *ZEB2* | Muscle |  |
| *IRAK2* | Liver, Muscle |  | *ZNF828* | Pancreas |  |
| *JAK2* | Adipose, Liver, Muscle, Pancreas |  |  |  |  |

[1] S. Xu, P. D. Witmer, S. Lumayag, et al., "MicroRNA (miRNA) transcriptome of mouse retina and identification of a sensory organ-specific miRNA cluster", *The Journal of biological chemistry.* vol. 282, no. 34, pp 25053-66, 2007.

[2] E. Saus, V. Soria, G. Escaramis, et al., "Genetic variants and abnormal processing of pre-miR-182, a circadian clock modulator, in major depression patients with late insomnia", *Human molecular genetics.* vol. 19, no. 20, pp 4017-25, 2010.

[3] L. Pedranzini, F. Mottadelli, S. Ronzoni, et al., "Differential cytogenomics and miRNA signature of the Acute Myeloid Leukaemia Kasumi-1 cell line CD34+38- compartment", *Leukemia research.* vol. 34, no. 10, pp 1287-95, 2010.

[4] O. Vaksman, H. T. Stavnes, J. Kaern, et al., "miRNA profiling along tumour progression in ovarian carcinoma", *Journal of cellular and molecular medicine.* vol. 15, no. 7, pp 1593-602, 2011.

[5] M. F. Segura, D. Hanniford, S. Menendez, et al., "Aberrant miR-182 expression promotes melanoma metastasis by repressing FOXO3 and microphthalmia-associated transcription factor", *Proceedings of the National Academy of Sciences of the United States of America.* vol. 106, no. 6, pp 1814-9, 2009.

[6] I. P. Pogribny, L. Muskhelishvili, V. P. Tryndyak and F. A. Beland, "The tumor-promoting activity of 2-acetylaminofluorene is associated with disruption of the p53 signaling pathway and the balance between apoptosis and cell proliferation", *Toxicology and applied pharmacology.* vol. 235, no. 3, pp 305-11, 2009.

[7] Q. Zhu, W. Sun, K. Okano, et al., "Sponge transgenic mouse model reveals important roles for the microRNA-183 (miR-183)/96/182 cluster in postmitotic photoreceptors of the retina", *The Journal of biological chemistry.* vol. 286, no. 36, pp 31749-60, 2011.

[8] V. Balatti, S. Maniero, M. Ferracin, et al., "MicroRNAs dysregulation in human malignant pleural mesothelioma", *Journal of thoracic oncology : official publication of the International Association for the Study of Lung Cancer.* vol. 6, no. 5, pp 844-51, 2011.

[9] M. D. Weston, M. L. Pierce, H. C. Jensen-Smith, et al., "MicroRNA-183 family expression in hair cell development and requirement of microRNAs for hair cell maintenance and survival", *Developmental dynamics : an official publication of the American Association of Anatomists.* vol. 240, no. 4, pp 808-19, 2011.

[10] H. Li, W. Kloosterman and D. M. Fekete, "MicroRNA-183 family members regulate sensorineural fates in the inner ear", *The Journal of neuroscience : the official journal of the Society for Neuroscience.* vol. 30, no. 9, pp 3254-63, 2010.

[11] A. V. Sirotkin, M. Laukova, D. Ovcharenko, et al., "Identification of microRNAs controlling human ovarian cell proliferation and apoptosis", *Journal of cellular physiology.* vol. 223, no. 1, pp 49-56, 2010.

[12] Z. Liu, J. Liu, M. F. Segura, et al., "MiR-182 overexpression in tumourigenesis of high-grade serous ovarian carcinoma", *The Journal of pathology.* vol. 228, no. 2, pp 204-15, 2012.

[13] J. Wang, J. Li, J. Shen, et al., "MicroRNA-182 downregulates metastasis suppressor 1 and contributes to metastasis of hepatocellular carcinoma", *BMC cancer.* vol. 12, no. pp 227, 2012.

[14] H. Hirata, K. Ueno, V. Shahryari, et al., "MicroRNA-182-5p promotes cell invasion and proliferation by down regulating FOXF2, RECK and MTSS1 genes in human prostate cancer", *PloS one.* vol. 8, no. 1, pp e55502, 2013.

[15] D. Yan, X. D. Dong, X. Chen, et al., "Role of microRNA-182 in posterior uveal melanoma: regulation of tumor development through MITF, BCL2 and cyclin D2", *PloS one.* vol. 7, no. 7, pp e40967, 2012.

[16] P. Guglielmelli, L. Tozzi, A. Pancrazzi, et al., "MicroRNA expression profile in granulocytes from primary myelofibrosis patients", *Experimental hematology.* vol. 35, no. 11, pp 1708-18, 2007.

[17] A. Yang, J. Ma, M. Wu, et al., "Aberrant microRNA-182 expression is associated with glucocorticoid resistance in lymphoblastic malignancies", *Leukemia & lymphoma.* vol. 53, no. 12, pp 2465-73, 2012.

[18] X. Xu, Z. Dong, Y. Li, et al., "The upregulation of signal transducer and activator of transcription 5-dependent microRNA-182 and microRNA-96 promotes ovarian cancer cell proliferation by targeting forkhead box O3 upon leptin stimulation", *The international journal of biochemistry & cell biology.* vol. 45, no. 3, pp 536-45, 2013.

[19] L. M. Friedman, A. A. Dror, E. Mor, et al., "MicroRNAs are essential for development and function of inner ear hair cells in vertebrates", *Proceedings of the National Academy of Sciences of the United States of America.* vol. 106, no. 19, pp 7915-20, 2009.

[20] T. Melkman-Zehavi, R. Oren, S. Kredo-Russo, et al., "miRNAs control insulin content in pancreatic beta-cells via downregulation of transcriptional repressors", *The EMBO journal.* vol. 30, no. 5, pp 835-45, 2011.

[21] X. Y. Chen, H. S. Zhang, T. C. Wu, et al., "Down-regulation of NAMPT expression by miR-182 is involved in Tat-induced HIV-1 long terminal repeat (LTR) transactivation", *The international journal of biochemistry & cell biology.* vol. 45, no. 2, pp 292-8, 2013.

[22] O. Giricz, P. A. Reynolds, A. Ramnauth, et al., "Hsa-miR-375 is differentially expressed during breast lobular neoplasia and promotes loss of mammary acinar polarity", *The Journal of pathology.* vol. 226, no. 1, pp 108-19, 2012.

[23] B. N. Hannafon, P. Sebastiani, A. de las Morenas, et al., "Expression of microRNA and their gene targets are dysregulated in preinvasive breast cancer", *Breast cancer research : BCR.* vol. 13, no. 2, pp R24, 2011.

[24] G. Li, C. Luna, J. Qiu, et al., "Alterations in microRNA expression in stress-induced cellular senescence", *Mechanisms of ageing and development.* vol. 130, no. 11-12, pp 731-41, 2009.

[25] S. Wu, S. Huang, J. Ding, et al., "Multiple microRNAs modulate p21Cip1/Waf1 expression by directly targeting its 3' untranslated region", *Oncogene.* vol. 29, no. 15, pp 2302-8, 2010.

[26] X. Peng, A. Vaishnav, G. Murillo, et al., "Protection against cellular stress by 25-hydroxyvitamin D3 in breast epithelial cells", *Journal of cellular biochemistry.* vol. 110, no. 6, pp 1324-33, 2010.

[27] Y. Q. Wang, R. D. Guo, R. M. Guo, et al., "MicroRNA-182 promotes cell growth, invasion, and chemoresistance by targeting programmed cell death 4 (PDCD4) in human ovarian carcinomas", *Journal of cellular biochemistry.* vol. 114, no. 7, pp 1464-73, 2013.

[28] H. Liu, Y. Wang, X. Li, et al., "Expression and regulatory function of miRNA-182 in triple-negative breast cancer cells through its targeting of profilin 1", *Tumour biology : the journal of the International Society for Oncodevelopmental Biology and Medicine.* vol. 34, no. 3, pp 1713-22, 2013.

[29] L. Jiang, P. Mao, L. Song, et al., "miR-182 as a prognostic marker for glioma progression and patient survival", *The American journal of pathology.* vol. 177, no. 1, pp 29-38, 2010.

[30] A. Schaefer, M. Jung, H. J. Mollenkopf, et al., "Diagnostic and prognostic implications of microRNA profiling in prostate carcinoma", *International journal of cancer Journal international du cancer.* vol. 126, no. 5, pp 1166-76, 2010.

[31] B. L. Pineles, R. Romero, D. Montenegro, et al., "Distinct subsets of microRNAs are expressed differentially in the human placentas of patients with preeclampsia", *American journal of obstetrics and gynecology.* vol. 196, no. 3, pp 261 e1-6, 2007.

[32] W. Q. Kong, R. Bai, T. Liu, et al., "MicroRNA-182 targets cAMP-responsive element-binding protein 1 and suppresses cell growth in human gastric adenocarcinoma", *The FEBS journal.* vol. 279, no. 7, pp 1252-60, 2012.

[33] L. Zhang, T. Liu, Y. Huang and J. Liu, "microRNA-182 inhibits the proliferation and invasion of human lung adenocarcinoma cells through its effect on human cortical actin-associated protein", *International journal of molecular medicine.* vol. 28, no. 3, pp 381-8, 2011.

[34] L. Song, L. Liu, Z. Wu, et al., "TGF-beta induces miR-182 to sustain NF-kappaB activation in glioma subsets", *The Journal of clinical investigation.* vol. 122, no. 10, pp 3563-78, 2012.

[35] I. K. Guttilla and B. A. White, "Coordinate regulation of FOXO1 by miR-27a, miR-96, and miR-182 in breast cancer cells", *The Journal of biological chemistry.* vol. 284, no. 35, pp 23204-16, 2009.

[36] G. Solda, M. Robusto, P. Primignani, et al., "A novel mutation within the MIR96 gene causes non-syndromic inherited hearing loss in an Italian family by altering pre-miRNA processing", *Human molecular genetics.* vol. 21, no. 3, pp 577-85, 2012.

[37] F. G. Rodriguez-Gonzalez, A. M. Sieuwerts, M. Smid, et al., "MicroRNA-30c expression level is an independent predictor of clinical benefit of endocrine therapy in advanced estrogen receptor positive breast cancer", *Breast cancer research and treatment.* vol. 127, no. 1, pp 43-51, 2011.

[38] M. S. Hildebrand, P. D. Witmer, S. Xu, et al., "miRNA mutations are not a common cause of deafness", *American journal of medical genetics Part A.* vol. 152A, no. 3, pp 646-52, 2010.

[39] W. C. Cho, A. S. Chow and J. S. Au, "Restoration of tumour suppressor hsa-miR-145 inhibits cancer cell growth in lung adenocarcinoma patients with epidermal growth factor receptor mutation", *European journal of cancer (Oxford, England : 1990).* vol. 45, no. 12, pp 2197-206, 2009.

[40] Y. Sun, R. Fang, C. Li, et al., "Hsa-mir-182 suppresses lung tumorigenesis through down regulation of RGS17 expression in vitro", *Biochemical and biophysical research communications.* vol. 396, no. 2, pp 501-7, 2010.

[41] A. M. Burroughs, Y. Ando, M. J. de Hoon, et al., "Deep-sequencing of human Argonaute-associated small RNAs provides insight into miRNA sorting and reveals Argonaute association with RNA fragments of diverse origin", *RNA biology.* vol. 8, no. 1, pp 158-77, 2011.

[42] T. J. Stark, J. D. Arnold, D. H. Spector and G. W. Yeo, "High-resolution profiling and analysis of viral and host small RNAs during human cytomegalovirus infection", *Journal of virology.* vol. 86, no. 1, pp 226-35, 2012.

[43] K. M. Kim, S. J. Park, S. H. Jung, et al., "miR-182 is a negative regulator of osteoblast proliferation, differentiation, and skeletogenesis through targeting FoxO1", *Journal of bone and mineral research : the official journal of the American Society for Bone and Mineral Research.* vol. 27, no. 8, pp 1669-79, 2012.

[44] W. Ishida, K. Fukuda, T. Higuchi, et al., "Dynamic changes of microRNAs in the eye during the development of experimental autoimmune uveoretinitis", *Investigative ophthalmology & visual science.* vol. 52, no. 1, pp 611-7, 2011.

[45] S. T. Mees, W. A. Mardin, C. Wendel, et al., "EP300--a miRNA-regulated metastasis suppressor gene in ductal adenocarcinomas of the pancreas", *International journal of cancer Journal international du cancer.* vol. 126, no. 1, pp 114-24, 2010.

[46] J. Krol, V. Busskamp, I. Markiewicz, et al., "Characterizing light-regulated retinal microRNAs reveals rapid turnover as a common property of neuronal microRNAs", *Cell.* vol. 141, no. 4, pp 618-31, 2010.

[47] A. K. Nagaraja, C. J. Creighton, Z. Yu, et al., "A link between mir-100 and FRAP1/mTOR in clear cell ovarian cancer", *Molecular endocrinology (Baltimore, Md).* vol. 24, no. 2, pp 447-63, 2010.

[48] X. R. Wang, X. M. Zhang, J. Zhen, et al., "MicroRNA expression in the embryonic mouse inner ear", *Neuroreport.* vol. 21, no. 9, pp 611-7, 2010.

[49] Y. Qu, W. C. Li, M. R. Hellem, et al., "MiR-182 and miR-203 induce mesenchymal to epithelial transition and self-sufficiency of growth signals via repressing SNAI2 in prostate cells", *International journal of cancer Journal international du cancer.* vol. 133, no. 3, pp 544-55, 2013.

[50] L. Wei, M. Wang, X. Qu, et al., "Differential expression of microRNAs during allograft rejection", *American journal of transplantation : official journal of the American Society of Transplantation and the American Society of Transplant Surgeons.* vol. 12, no. 5, pp 1113-23, 2012.

[51] S. S. Myatt, J. Wang, L. J. Monteiro, et al., "Definition of microRNAs that repress expression of the tumor suppressor gene FOXO1 in endometrial cancer", *Cancer research.* vol. 70, no. 1, pp 367-77, 2010.

[52] A. B. Stittrich, C. Haftmann, E. Sgouroudis, et al., "The microRNA miR-182 is induced by IL-2 and promotes clonal expansion of activated helper T lymphocytes", *Nature immunology.* vol. 11, no. 11, pp 1057-62, 2010.

[53] A. Dolganiuc, J. Petrasek, K. Kodys, et al., "MicroRNA expression profile in Lieber-DeCarli diet-induced alcoholic and methionine choline deficient diet-induced nonalcoholic steatohepatitis models in mice", *Alcoholism, clinical and experimental research.* vol. 33, no. 10, pp 1704-10, 2009.

[54] H. Bruchova, M. Merkerova and J. T. Prchal, "Aberrant expression of microRNA in polycythemia vera", *Haematologica.* vol. 93, no. 7, pp 1009-16, 2008.

[55] X. R. Wang, X. M. Zhang, J. Du and H. Jiang, "MicroRNA-182 regulates otocyst-derived cell differentiation and targets T-box1 gene", *Hearing research.* vol. 286, no. 1-2, pp 55-63, 2012.
